# Dataset for the life cycle assessment of the high speed rail network in Spain

**DOI:** 10.1016/j.dib.2021.107006

**Published:** 2021-04-12

**Authors:** Andoni Kortazar, Gorka Bueno, David Hoyos

**Affiliations:** a Department of Public Policy and Economic History, Faculty of Business and Economics, Araba section, Research Group on Ecological Economics & Political Ecology, University of the Basque Country UPV/EHU, Member of EKOPOL, Spain; b Department of Electronics Engineering, Faculty of Engineering, Research Group on Ecological Economics & Political Ecology, University of the Basque Country UPV/EHU, Member of EKOPOL, Spain; c Department of Applied Economics III (Econometrics & Statistics), Faculty of Business and Economics, Bilbao, Research Group on Ecological Economics & Political Ecology, University of the Basque Country UPV/EHU, Member of EKOPOL, Spain

**Keywords:** High speed train, Sustainable mobility, Life cycle assessment (LCA), Environmental impact, Energy consumption

## Abstract

A life cycle assessment (LCA) of the Spanish high speed rail (HSR) network in service in 2016 (2583 km) was conducted. Life cycle inventory (LCI) data related to the construction and maintenance phases of the infrastructure was collected using Google Earth tool, and complemented with data obtained from the LCA carried out by Tuchschmid et al. [1]. LCI data associated with the operation phase of the infrastructure was built on available fragmentary data on passenger movements for the year 2016 [2–4], processed with a python algorithm to estimate the transport service provided by the infrastructure. Environmental impacts for transport modes were obtained from Ecoinvent v3.7 database [5,6] and processed with openLCA software [7]. Life cycle impact assessment (LCIA) results gathered in the dataset include Global Warming (GWP100a), Cumulative Energy Demand and total emissions for PM10, SO_2_, NO_X_ and NMVOC. This dataset presents a detailed description of the Spanish HSR network, including the length of each item (bridges, tunnels, earthworks, railway tracks), and a robust estimation of passenger transport over the infrastructure for year 2016. The LCI data presented in this paper support the original research done on whether the construction of Spanish HSR network infrastructure is justified in terms of reducing environmental impacts and energy consumption [8], and may be used as a baseline for future studies on transport economics.

## Specifications Table

**Table:** 

Subject	Transportation and environment
Specific subject area	Life Cycle Assessment and rail transport
Type of data	Text, tables, figures, Excel spreadsheet
How data were acquired	Raw data was acquired from primary and secondary data sources, including: (i) Google Earth tool, providing visual information related to the elements in the HSR network; (ii) public reports on passenger movements on the network in 2016; (iii) LCA studies on other HSR infrastructures; and (iv) Ecoinvent v3.7 commercial database.
Data format	Raw and analysed data
Parameters for data collection	The network considered in the LCI was the Spanish HSR in operation in 2016. Primary data for the calculation of passenger transport were passenger arrivals and departures for every HSR station in 2016, and passenger movements for some specific connections in 2016. High-speed services considered were AVE, ALVIA, AVANT and AV-City. Alternative scenarios were analysed considering different combinations of occupancy rates and propulsion technologies for private cars, electricity mixes and future HSR transport demand.
Description of data collection	The description of the infrastructure elements of the HSR network was performed using Google Earth and its aerial perspective. Complementary data for the LCI of the construction and maintenance phases was obtained from Tuchschmid et al. [1]. Passenger transport in the network was calculated for year 2016 with a python algorithm applied to primary data of passenger arrivals and departures for every HSR station in 2016, provided by El País [3] and El Independiente [2] journals, and to passenger movements in some specific connections, provided by Fundación de los Ferrocarriles Españoles [9]. Environmental impacts and material flows for modes of transport were collated from the Ecoinvent v3.7 database [5,6] processed with openLCA v1.10 [7]. LCIA results were obtained for Global Warming (GWP100a) and Cumulative Energy Demand, along with total emissions for PM10, SO_2_, NO_X_ and NMVOC.
Data source location	Layout of the four high-speed network corridors in Spain and all the network stations with high-speed services. Institution: ADIF Regions: Madrid, Castilla y León, Aragón, Catalonia, Castilla La Mancha, Valencia, Andalusia. Country: Spain GPS coordinates of main stations: Madrid-Chamartín: 40.47247577338163, −3.682445817927162. Madrid-Atocha: 40.406481189023786, −3.6901169354945793. Sevilla: 37.39218125602973, −5.976725622061928. Valencia: 39.46019137048506, −0.3801866364308562. Barcelona: 41.39110863604537, 2.147779823223015. León: 42.5949441654377, −5.581576017959486. Malaga: 36.72024142175778, −4.437932014354047. Alicante: 38.34533185646225, −0.49355176317645394. Zaragoza: 41.65606748915106, −0.9044368527123184. Primary data sources (secondary data for this work): LCI of the construction and maintenance phases from Tuchschmid et al. [1]. Passenger traffic data from El Independiente [2], El País [3] and Fundación de los Ferrocarriles Españoles [9].
	Percentages of shifted transport from other modes from Betancor and Llobet [4]. LCI data for modes of transport from the Ecoinvent v3.7 database [5,6].
Data accessibility	Data are available within the article and detailed supplementary information is also provided through a repository. Repository name: Mendeley Data Data identification number: http://dx.doi.org/10.17632/5f2szm73s7.2 Direct URL to data: http://dx.doi.org/10.17632/5f2szm73s7.2
Related research article	Andoni Kortazar, Gorka Bueno, David Hoyos “Environmental balance of the High Speed Rail network in Spain: a Life Cycle Assessment approach” Research in Transportation Economics https://doi.org/10.1016/j.retrec.2021.101035

## Value of the Data

• This dataset gathers a precise geographical description of the Spanish HSR network infrastructure, including the measurement of each item, which can be crucial to different studies of the Spanish HSR.
• The dataset provides necessary detailed life cycle inventories to perform the life cycle assessment of the Spanish HSR network but can be used as a baseline for researchers to perform a LCA of other HSR networks.
• This dataset may be used in future academics studies on transport economics, e.g. to calculate environmental footprints or economic profiles, and for any stakeholder interested in the environmental sustainability of HSR lines.
• This dataset provides with passenger transport estimates over Spanish corridors in 2016 measured in passenger-kilometre.

## 1 Data Description

This dataset provides all primary and secondary data used to build the LCA of the entire Spanish HSR network in operation in 2016. The LCI data presented support the original research done on whether the construction of that infrastructure is justified in terms of reducing environmental impacts and energy consumption, under different scenarios [8].

Table 1 provides background data related to the reference scenario and the alternative scenarios. This background data includes not only the characterisation of the electricity mix that powers the high-speed rail infrastructure, but also the transport characteristics of the modes from which the infrastructure shifts passengers, in a situation where the HSR infrastructure is not in operation.

**Table 1:** Background data related to the reference scenario (Baseline) and the alternative scenarios (S1–5).

	BaselineScenario	S1	S2	S3	S4	S5
Description of the scenario	General conditions (transport demand, shifted transport from other modes, electricity mix, vehicle occupancy rates) are those documented for Spain in year 2016, and are maintained throughout the lifetime of the infrastructure.	Private vehicles present occupancy rates expected for Spain in 2020	Private vehicles double occupancy rates in comparison to 2016	HSR transport demand doubles in all corridors in comparison to 2016 transport demand	All private cars are electric	All private cars are electrified and electric propulsion is powered with electricity of renewable origin (cars and electric trains)
Electricity mix	Spain 2017; as specified by Ecoinvent v3.7 for Spain in 2017: market for electricity, high voltage (ES) market for electricity, low voltage (ES)					100% renewable; electricity from fossil fuels and nuclear removed from the mix
HSR demand	Transport demand in the HSR line in year 2016 (Baseline Scenario demand)			Transport demand doubles in comparison with the Baseline Scenario	Baseline Scenario demand	
Shifted transport from other modes	Percentages of shifted transport from other modes follow information provided by Betancor and Llobet [4]					
Passenger car occupancy rate	1.68 passengers per car; average occupancy rate in Spain in 2014 [10]	2.52 passengers per car; estimated for Spain in 2020 by Adra et al. [11]	3.36 passengers per car; occupancy rate doubles in comparison with the Baseline Scenario	1.68 passengers per car; average occupancy rate in Spain in 2014 [10]		3.36 passengers per car; occupancy rate doubles in comparison with the Baseline Scenario
Car Propulsion	56% of cars are diesel and 44% are petrol powered, statistical data from [12] Vehicle characteristics as specified by Ecoinvent v3.7 Diesel vehicle: transport, passenger car, medium size, diesel, EURO 5 (RER) Petrol vehicle: transport, passenger car, medium size, petrol, EURO 5 (RER)				Electric characteristics as specified by Ecoinvent v3.7 vehicle: transport, passenger car, electric (GLO) electricity: market for electricity, low voltage (ES)	Electric propulsion with renewable electricity Same as S4; electricity from fossil fuels and nuclear removed from the mix
HSR operationld	as specified by Ecoinvent v3.7 transport, passenger train, high-speed (DE); with electricity: market for electricity, high voltage (ES)					Same as others except electricity for HSR and conventional trains, in which supply from fossil fuels and nuclear is removed from the mix
Characteristics of other transport modes (aeroplane, coach, conventional train)	Vehicle characteristics as specified by Ecoinvent v3.7 for the following transport modes: Aeroplane: transport, passengers, aircraft, very short haul (GLO) Coach: transport, passenger coach (CH) Conventional train: transport, passenger train (DE) ; with electricity: market for electricity, high voltage (ES)					

Data is structured in three parts: the first part (1.1) describes the LCI of the infrastructure's construction and maintenance phase; the second part (1.2) gathers the LCI of the operation phase and the third one (1.3) presents the LCIA data.

### 1.1 LCI on the construction and maintenance phase

The Spanish HSR network consists of four main corridors that connect different regions with the state capital, Madrid: Madrid-Catalonia, Madrid-León (Northern corridor), Madrid-Levante and Madrid-Andalusia. In total, 2583 km were in service in the year 2016.

A detailed description of each of the four corridors of the HSR network, including the length of each item (bridges, tunnels, earthworks, railway tracks, passageways, forks, stations), is gathered in the “Layout” tab of each of the four corridors’ attached Excel files (“XX CORRIDOR Ecoinvent 3.7.xlsx”, where XX is ANDALUSIA, CATALONIA, LEVANTE or NORTHERN) included in SM01 (SM means Supplementary Material).

The LCI for the construction and maintenance phase of each of the HSR corridors is gathered in SM02. Tables 1–8 in the “SM02.docx” file gather the LCI of bridges (Table 1), earthworks (Table 2), tunnels (Table 3), sleepers and ballasts (Table 4), rails (Table 5), mast, catenary and overhead wiring (Table 6), signals and communication systems (Table 7) and construction and maintenance of railway buildings (Table 8) in the four corridors of the Spanish HSR. Table 2 summarises the LCI for the whole network. LCI data provided by Tuchschmid et al. [1] in relation to Cumulative Energy Demand, CO_2_, PM10, SO_2_, NO_X_ and NMVOC emissions flows related to the construction and maintenance of items is also shown, in terms per km and year (or per unit and year, where applicable).

**Table 2:** LCI of Spanish HSR network elements and flows related to construction and maintenance, per km and year (or per unit and year, where applicable), based on Tuchschmid et al. [1].

	Andalusia corridor	Northern corridor	Catalonia corridor	Levante corridor	CO_2_	CED	PM_10_	SO_2_	NO_X_	NMVOC
	km	km	km	km	kg·y^−1^·km^−1^	MJ-equ·y^−1^·km^−1^	kg·y^−1^·km^−1^	kg·y^−1^·km^−1^	kg·y^−1^·km^−1^	kg·y^−1^·km^−1^
Viaduct, single track	1.556	2.551	0.581	1.82	154,925	1,485,081	154	183	319	60
Viaduct, double track	16.663	11.769	48.53	25.518	258,209	2,475,135	256	305	532	100
Small bridge, concrete, single track	0	2.361	0.713	0	67,534	646,981	67	80	139	26
Small bridge, concrete, double track	4.42	4.229	16.35	9.515	112,557	1,078,302	112	133	232	44
Iron bridge, single track	0	0	0.111	0	136,412	2,246,780	468	352	332	54
Earthwork, single track renewal of existing lines	0	40.645	20.7	0	1652	31,815	1.63	2.77	18.29	2.58
Earthwork, double track renewal of existing lines	0	44.805	0	0	2753	53,025	2.72	4.61	30.48	4.3
Earthwork, single track new constructed lines	0	50.551	72.66	0	5993	109,103	6	10	68	10
Earthwork, double track new constructed lines	646.8	309.2	789.6	607.92	9791	176,878	10.51	15.94	110.78	16.11
Open pit, single track	0	0.068	0.112	0	285.35	3037.29	0.43	0.42	0.63	0.10
Open pit, double track	0.547	0.278	4.14	0.408	475.58	5062.16	0.72	0.70	1.06	0.17
Mining, single track	14.6	74.114	7.5	14.458	169.61	1591.13	0.13	0.20	0.37	0.05
Mining, double track	31.346	9.33	83.594	44.381	282.69	2651.88	0.22	0.34	0.63	0.09
Concrete sleeper, single track	0	91.196	93.36	0	5184	84,827	6.03	9.77	17.32	3.13
Concrete sleeper, double track	646.8	354.005	789.6	607.92	10,340	169,058	12.02	19.48	34.53	6.23
Rail UIC 60, single track	0	91.196	93.36	0	6.09	96.97	0.0182	0.0134	0.0138	0.0021
Rail UIC 60, double track	646.8	354.005	789.6	607.92	12.18	193.94	0.0365	0.0268	0.0276	0.0043
Wire of catenary, single track	0	91.196	93.36	0	0.353	6.938	0.0039	0.0184	0.0040	0.0007
Wire of catenary, double track	646.8	354.005	789.6	607.92	0.706	13.875	0.0078	0.0369	0.0080	0.0014
Mast & overhead wiring, iron, single track	0	91.196	91.46	0	1.235	21.974	0.0031	0.0035	0.0028	0.0006
Mast & overhead wiring, iron, double track	600.307	307.272	696.154	548.673	2.469	43.948	0.0062	0.0071	0.0056	0.0012
Overhead wiring, single track, tunnel	14.6	74.182	7.612	14.458	0.536	10.413	0.0009	0.0019	0.0011	0.0002
Overhead wiring, double track, tunnel	31.893	9.608	87.734	44.789	1.071	20.826	0.0019	0.0039	0.0023	0.0004
Signals per km single track	0	91.196	93.36	0	33	548	0.09	0.08	0.08	0.02
Signals per km double track	646.8	354.005	789.6	607.92	56	914	0.14	0.13	0.14	0.03
Cable for telecommunication per km single track	0	91.196	93.36	0	380	11,632	3	14	3	1
Cable for telecommunication per km double track	646.8	354.005	789.6	607.92	634.47	19,400.9	4.38	23.48	5.26	1.51
Cable drain per km single track	0	91.196	93.36	0	295.21	2634.42	0.07	0.23	0.68	0.16
Cable drain per km double track	646.8	354.005	789.6	607.92	491.81	4388.95	0.12	0.38	1.13	0.26

	Unit	Unit	Unit	Unit	kg·y^−1^·unit^−1^	MJ-eq·y^−1^·unit^−1^	kg·y^−1^·unit^−1^	kg·y^−1^.unit^−1^	kg·y^−1^·unit^−1^	kg·y^−1^·unit^−1^

Junction for intercity trains	10	5	11	6	164,714	1,310,519	115	165	310	54
Junction for local trains	0	2	0	0	68,142	539,044	47	68	128	22
Railway control centre, building	10	7	11	6	226.96	3886.42	0.62	0.54	0.51	0.14
Railway control centre, electrical installations	10	7	11	6	965.43	20,254.63	3.05	5.48	2.78	0.83

**Table 3:** LCI for the operation phase of the four corridors, in the baseline scenario. Transport is given annually and measured in passenger-kilometres (pkm).

TRANSPORT MODE	Andalusia corridor	Northern corridor	Catalonia corridor	Levante corridor	Global Warming	CED MJ·pkm^−1^	PM10 g·pkm^−1^	SO_2_ g·pkm^−1^	NO_X_ g·pkm^−1^	NMVOC
	pkm	pkm	pkm	pkm	gCO_2_eq·pkm^−1^					g·pkm^−1^
Passenger aircraft, very short haul	1,559,884,843	0	1,275,421,783	742,225,091	159.37	2.41	0.04	0.22	0.72	0.10
Passenger coach	69,328,215.26	29,151,271.76	104,414,512.2	32,987,781.8	49.44	0.82	0.03	0.05	0.47	0.05
Passenger car mix (56% diesel, 44% petrol, 1,68 passengers per car)	415,969,291.6	174,907,630.6	1,264,198,270	247,408,364	187.39	2.86	0.11	0.31	0.46	0.17
Passenger train, Spanish electricity mix	901,266,798.4	204,058,902.3	1,800,447,020	494,816,727	54.65	1.18	0.05	0.19	0.31	0.03
Passenger train, high-speed, Spanish electricity mix, (HSR infrastructure excluded)	3,466,410,763	583,025,435.2	5,082,583,531	1,649,389,091	32.91	0.86	0.03	0.15	0.10	0.01

**Table 4:** LCIA methods considered in the assessment and elementary flows for which final net balances have been analysed.

Impact category	
Global Warming (100a)	LCIA method: CML-IA (baseline) [13] Global warming potential for a 100-year time horizon (GWP100) for each greenhouse gas emission to the air; values reported by IPCC (2013) Unit: kilogram CO_2_-eq
Total Cumulative Energy Demand	LCIA method: Cumulative Energy Demand (CED) Based on the method published by Ecoinvent [14] Normalization and weighting set: Cumulative energy demand Unit: MJ
Elementary flow	
Particulate Matter (PM10)	Emissions to air of particulates (particulate matter) measuring less than 10 µm in diameter. Unit: kg Related impact category: Human toxicity (CML-IA baseline)
Sulphur Dioxide (SO_2_)	Emissions to air of sulphur dioxide. Unit: kg Related impact categories: Acidification, Human toxicity, Photochemical oxidation (CML-IA baseline)
Non-Methane Volatile Organic Compounds (NMVOC)	Emissions to air of non-methane volatile organic compounds (NMVOC). Unit: kg Related impact categories: Terrestrial and aquatic ecotoxicities, Human toxicity, Photochemical oxidation, Ozone layer depletion (CML-IA non-baseline)
Nitrogen Oxides (NO_x_)	Emissions to air of nitrogen oxides. Unit: kg Related impact categories: Acidification, Eutrophication, Human toxicity (CML-IA baseline); Photochemical oxidation (CML-IA non-baseline)

**Table 5:** Data related to the processing of LCIA results and total emissions of selected elementary fluxes for the Andalusia corridor.

	CO_2_eq	CED	PM10	SO_2_	NO_X_	NMVOC
Andalusia corridor	kt·y^−1^	TJ·y^−1^	t·y^−1^	t·y^−1^	t·y^−1^	t·y^−1^
Infrastructure Construction & Maintenance	41.94	572.14	65.90	106.13	164.14	26.65
Operation (5.36 million passengers) (Baseline)	114.08	2981.00	117.57	531.87	357.71	21.94
Shifted transport (Baseline)	379.23	6066.28	158.68	643.08	1622.91	249.84
Net Impact (Baseline)	−223.21	−2513.14	24.80	−5.08	−1101.06	−201.25
Net Impact (S1, 2.52 passengers/vehicle)	−197.22	−2116.42	39.88	38.53	−1036.91	−178.24
Net Impact (S2, 3.36 passengers/vehicle)	−184.23	−1918.06	47.42	60.34	−1004.83	−166.74
Net Impact (S3, transport in HSR doubles)	−488.35	−5598.42	−16.31	−116.29	−2366.26	−429.14
Net Impact (S4, electric mobility)	−183.83	−2152.06	19.53	−23.53	−1029.10	−162.49
Net Impact (S5, high occup. ren. elec. mobility)	−223.98	−2706.52	5.19	−257.11	−1153.26	−152.80

Annual transport for null impact (Baseline, Mp)	0.85	0.99	8.59	5.11	0.70	0.63
Annual transport for null impact (S1, Mp)	0.94	1.14	13.57	8.41	0.73	0.70
Annual transport for null impact (S2, Mp)	0.99	1.23	19.11	12.42	0.75	0.74
Induced transport for null impact (S3,%)	69.73	54.22	19.37	22.69	76.97	88.00
Annual transport for null impact (S4, Mp)	1.00	1.13	7.62	4.39	0.74	0.76
Annual transport for null impact (S5, Mp)	0.85	0.94	5.82	1.57	0.67	0.80

Years required for compensation (Baseline, years)	9	11	96	57	8	7
Years required for compensation (S1, years)	11	13	152	94	8	8
Years required for compensation (S2, years)	11	14	214	139	8	8
Years required for compensation (S3, years)	5	6	48	29	4	4
Years required for compensation (S4, years)	11	13	85	49	8	8
Years required for compensation (S5, years)	9	10	65	18	7	9

**Table 6:** Data related to the processing of LCIA results and total emissions of selected elementary fluxes for the Northern corridor.

	CO_2_eq	CED	PM10	SO_2_	NO_X_	NMVOC
Northern corridor	kt·y^−1^	TJ·y^−1^	t·y^−1^	t·y^−1^	t·y^−1^	t·y^−1^
Infrastructure Construction & Maintenance	34.61	432.17	47.31	76.30	117.06	19.02
Operation (1.30 million passengers) (Baseline)	19.19	501.38	19.77	89.46	60.16	3.69
Shifted transport (Baseline)	45.37	765.56	30.84	94.65	156.84	37.01
Net Impact (Baseline)	8.42	167.99	36.24	71.11	20.39	−14.29
Net Impact (S1, 2.52 passengers/vehicle)	19.35	334.80	42.58	89.45	47.36	−4.62
Net Impact (S2, 3.36 passengers/vehicle)	24.81	418.21	45.75	98.62	60.85	0.21
Net Impact (S3, transport in HSR doubles)	−17.76	−96.19	25.17	65.92	−76.29	−47.61
Net Impact (S4, electric mobility)	24.98	319.82	34.03	63.35	50.64	2.00
Net Impact (S5, high occup. ren. elec. mobility)	26.45	385.85	40.10	59.28	55.73	7.75

Annual transport for null impact (Baseline, Mp)	1.73	2.14	5.60	19.25	1.59	0.75
Annual transport for null impact (S1, Mp)	2.97	5.81	13.11	−7.60	2.20	1.05
Annual transport for null impact (S2, Mp)	4.63	40.55	39.80	−4.48	2.73	1.32
Induced transport for null impact (S3,%)	43.70	34.40	1.43	5.62	47.02	75.03
Annual transport for null impact (S4, Mp)	7.08	7.70	7.00	12.72	2.58	0.99
Annual transport for null impact (S5, Mp)	5.56	12.22	8.60	5.87	2.50	2.21

Years required for compensation (Baseline, years)	79	98	256	882	73	34
Years required for compensation (S1, years)	136	266	600	−348	101	48
Years required for compensation (S2, years)	212	1 858	1 823	−205	125	61
Years required for compensation (S3, years)	40	49	128	441	36	17
Years required for compensation (S4, years)	216	231	214	354	106	67
Years required for compensation (S5, years)	255	560	394	269	115	101

**Table 7:** Data related to the processing of LCIA results and total emissions of selected elementary fluxes for the Catalonia corridor.

	CO_2_eq	CED	PM10	SO_2_	NO_X_	NMVOC
Catalonia corridor	kt·y^−1^	TJ·y^−1^	t·y^−1^	t·y^−1^	t·y^−1^	t·y^−1^
Infrastructure Construction & Maintenance	73.81	927.77	103.72	161.62	254.79	41.69
Operation (5.76 million passengers) (Baseline)	167.27	4370.85	172.39	779.85	524.49	32.17
Shifted transport (Baseline)	543.71	8900.62	288.55	1017.30	2101.56	393.85
Net Impact (Baseline)	−302.64	−3602.00	−12.44	−75.83	−1322.28	−319.98
Net Impact (S1, 2.52 passengers/vehicle)	−223.67	−2396.30	33.39	56.71	−1127.32	−250.08
Net Impact (S2, 3.36 passengers/vehicle)	−184.19	−1793.45	56.30	122.98	−1029.84	−215.13
Net Impact (S3, transport in HSR doubles)	−679.08	−8131.77	−128.61	−313.28	−2899.36	−681.66
Net Impact (S4, electric mobility)	−182.98	−2504.61	−28.44	−131.91	−1103.59	−202.21
Net Impact (S5, high occup. ren. elec. mobility)	−186.40	−2256.37	6.21	−234.70	−1110.02	−161.92

Annual transport for null impact (Baseline, Mp)	1.13	1.18	5.14	3.92	0.93	0.66
Annual transport for null impact (S1, Mp)	1.43	1.61	8.49	8.87	1.06	0.82
Annual transport for null impact (S2, Mp)	1.65	1.96	12.59	24.08	1.14	0.93
Induced transport for null impact (S3,%)	100.81	78.02	49.46	34.77	102.55	144.44
Annual transport for null impact (S4, Mp)	1.65	1.56	4.52	3.17	1.08	0.98
Annual transport for null impact (S5, Mp)	1.63	1.68	6.12	2.35	1.07	1.18

Years required for compensation (Baseline, years)	12	12	54	41	10	7
Years required for compensation (S1, years)	15	17	88	92	11	9
Years required for compensation (S2, years)	17	20	131	251	12	10
Years required for compensation (S3, years)	6	6	27	20	5	3
Years required for compensation (S4, years)	17	16	47	33	11	10
Years required for compensation (S5, years)	17	17	64	24	11	12

**Table 8:** Data related to the processing of LCIA results and total emissions of selected elementary fluxes for the levante corridor.

	CO_2_eq	CED	PM10	SO_2_	NO_X_	NMVOC
Levante corridor	kt·y^−1^	TJ·y^−1^	t·y^−1^	t·y^−1^	t·y^−1^	t·y^−1^
Infrastructure Construction & Maintenance	46.33	603.75	68.09	108.17	169.32	27.60
Operation (2.71 million passengers) (Baseline)	54.28	1418.42	55.94	253.07	170.20	10.44
Shifted transport (Baseline)	193.32	3106.05	84.41	333.85	815.25	129.18
Net Impact (Baseline)	−92.71	−1083.88	39.63	27.40	−475.73	−91.15
Net Impact (S1, 2.52 passengers/vehicle)	−77.26	−847.92	48.60	53.34	−437.57	−77.47
Net Impact (S2, 3.36 passengers/vehicle)	−69.53	−729.94	53.08	66.31	−418.49	−70.63
Net Impact (S3, transport in HSR doubles)	−231.75	−2771.51	11.16	−53.38	−1120.78	−209.89
Net Impact (S4, electric mobility)	−69.29	−869.12	36.50	16.42	−432.93	−68.10
Net Impact (S5, high occup. ren. elec. mobility)	−83.38	−1039.34	34.42	−72.64	−476.04	−61.44

Annual transport for null impact (Baseline, Mp)	0.90	0.97	6.49	3.63	0.71	0.63
Annual transport for null impact (S1, Mp)	0.10	0.11	0.95	0.54	0.08	0.07
Annual transport for null impact (S2, Mp)	0.11	0.12	1.23	0.70	0.08	0.08
Induced transport for null impact (S3,%)	63.14	49.05	1.92	15.35	71.24	82.74
Annual transport for null impact (S4, Mp)	1.09	1.11	5.85	3.20	0.76	0.78
Annual transport for null impact (S5, Mp)	0.97	1.00	5.49	1.62	0.71	0.84

Years required for compensation (Baseline, years)	20	21	144	80	16	14
Years required for compensation (S1, years)	22	25	210	118	17	16
Years required for compensation (S2, years)	24	27	272	155	17	17
Years required for compensation (S3, years)	10	11	72	40	8	7
Years required for compensation (S4, years)	24	25	129	71	17	17
Years required for compensation (S5, years)	21	22	121	36	16	19

### 1.2 LCI of the operation phase

The LCI of the operation phase requires an accurate estimation of passenger transport over the network. This, in turn, requires knowledge of passenger movements between stations. Since the LCA is orientated towards the evaluation of the implementation of the HSR infrastructure, it is also necessary to compute the passenger transport displaced from other modes to the HSR network, in comparison with an alternative scenario in which the infrastructure is not operative.

In the SM03 excel file (“SM03.xlsx”), the “HSR station Traffic 2016″ tab presents the passenger arrives and departs in each station of the Spanish HSR in 2016. This data was provided by Galán et al. [3] and by García [2]. The “Specific connections” tab presents data of passenger movements in some specific connections provided by Fundación de los Ferrocarriles Españoles [9]. The “Shifted transport” tab from the same excel file presents the percentages for shifted transport from other transport modes to the HSR, for each of the corridors, obtained from Betancor and Llobet [4].

The SM04 file (“SM04.docx”) presents four tables, one for each corridor, with the matrix of distances between each pair of stations (Table 1 for the Andalusia corridor, Table 2 for the Northern corridor, Table 3 for the Catalonia corridor and Table 4 for the Levante corridor). Each table also provides the number of annual passenger arrivals and departures in each HSR station.

The SM06 file (“SM06.docx”) provides the estimation for the passenger transport in the HSR that is coherent with the data gathered in SM03 and SM04. In particular, this file provides a table for each corridor with a matrix of average passengers up and down in each HSR station (Table 1 for the Nothern corridor, Table 2 for the Andalusia corridor, Table 3 for the Levante corridor and Table 4 for the Catalonia corridor). Statistical results for the passenger transport estimation in each corridor is also graphically provided in Figs. 1–4 of the same file station (Fig. 1 for the Nothern corridor, Fig. 2 for the Andalusia corridor, Fig. 3 for the Levante corridor and Fig. 4 for the Catalonia corridor).

The SM07 file (“SM07.xlsx”) provides the LCI data for the transport modes considered in the operation phase of the HSR, obtained from the Ecoinvent v3.7 database [5,6] and processed with openLCA software v1.10 [7].

Based on data from SM03, SM04, SM06 and SM07, Table 3 presents the LCI of the operation phase in each corridor of the Spanish HSR network, in the Baseline Scenario. The LCI for the operation phase in the alternative scenarios (S1–5) is gathered in the SM08 file (“SM08.docx”). Table 1 from this document gathers the LCI for the operation phase in the alternative Scenario 1, Table 2 for alternative Scenario 2, Table 3 for alternative Scenario 3, Table 4 for alternative Scenario 4 and Table 5 for alternative Scenario 5.

### 1.3 LCIA and total emissions data

LCIA results gathered in the dataset include Global Warming (GWP100a) and Cumulative Energy Demand. Total emissions to air of the elementary flows PM10, SO_2_, NO_X_ and NMVOC have also been considered in the analysis. Table 4 provides information on the LCIA methods considered and the elementary flows for which final net balances have been analysed.

Tables 5–8 present data related to the processing of LCIA results and total emissions of selected elementary fluxes for the four corridors of the Spanish HSR, into three separate blocks. The first block presents the environmental impacts linked to the construction and maintenance phase, the operation phase and to the transport shifted from other modes for the Baseline Scenario, and the net environmental impacts for all the scenarios explored. These results are provided in a yearly basis, considered that the lifetime of the infrastructure is 60 years. The second block presents the quantity of annual passengers (in millions) needed in each corridor, in each scenario, to reach a null net environmental impact. For scenario S3, the value shown in the table is the percentage of induced transport that provides null environmental impact (in S3, transport demand exactly doubles that estimated for 2016). The third block presents the years of HSR operation needed to compensate the environmental impacts associated to the construction and maintenance of the infrastructure. Complete and detailed results are available in the excel files included in SM01.

Table 9 presents GHG equivalent emissions linked to infrastructure construction and maintenance for each corridor in a yearly basis, and for each passenger-kilometre (pkm) travelled in each corridor, in the Baseline Scenario (assuming that transport demand in 2016 is maintained throughout the lifetime of the infrastructure).

**Table 9:** GHG emissions linked to infrastructure construction and maintenance per passenger-kilometre, in each corridor.

Corridor	Length(km)	Transport (million pkm)	Annual GHG emissions linked to infrastructure construction and maintenance, per km of corridor (t CO_2_·km^−1^·y^−1^)	Annual GHG emissions linked to infrastructure construction and maintenance in each corridor (t CO_2_·y^−1^)	GHG emissions linked to infrastructure construction and maintenance per pkm, in each corridor (g CO_2_/pkm)
Andalusia	647	3466	64.85	41,942	12.10
Northern	445	583	77.78	34,606	59.36
Catalonia	883	5083	83.59	73,809	14.52
Levante	608	1649	76.21	46,331	28.09
Total	2583	10,781		196,689	18.24

## 2 Experimental Design, Materials and Methods

The purpose of the work that gathered this dataset was to carry out a comparative LCA of the Spanish HSR network, in order to verify whether its construction is justified in terms of reducing environmental impacts and energy consumption. The goal of the LCA was set to obtain and present the inventory data linked to the life-cycle of the infrastructure, also considering the environmental contribution of shifted transport from other modes to the HSR. The scope of the study was the Spanish HSR transport system constructed and in operation in year 2016. The functional unit was the passenger transport service provided by the entire HSR network in one year of operation, which includes the AVE, ALVIA, AVANT and AV-City high-speed services in Spain. Primary data presented in the previous section was processed in a modelling that is described in the remainder of this section.

The net environmental impact (NetEI) linked to the functional unit, in comparison with a transport system in which the HSR is not operative, is calculated following Eq. (1):

(1) $$NetEI=\sum EI_{Construction\;\&\;Maintenance}^{HSR}+\sum EI_{Operation}^{HSR}-\sum_{i}EI_{i\to HSR}^{i}$$

where $EI_{Construction\;\&\;Maintenance}^{HSR}$ denotes the construction and maintenance impacts of the infrastructure, considering that they are evenly distributed along its lifetime (60 years) [15]; $EI_{Operation}^{HSR}$ denotes the impacts related to the annual operation of the HSR infrastructure; and $EI_{i\to HSR}^{i}$ denotes the impacts linked to the annual operation of alternative transport modes –referenced with the *i* subscript, i.e. aeroplane, conventional train, coach and private car–, from which transport is shifted to the HSR [8].

Detailed information was needed on every kilometre of the Spanish HSR constructed and in operation in year 2016, for the compilation of the inventory of the construction and maintenance phase. Faced with the impossibility of obtaining the construction plans of every line in the network, the data was obtained using the Google Earth tool. A complete aerial review of each of the lines that make up the network was carried out, and all the measurements of the different items were recorded. The environmental impacts associated with the construction and maintenance of the HSR network were calculated applying to each item of the infrastructure the corresponding impact coefficient following Tuchschmid et al. [1]. The calculation for each corridor is gathered in the “CONSTRUCTION IMPACTS MODELLING” tab of the corresponding Excel file included in SM01.

The traffic data gathered for year 2016 (available in the attached files SM03 and SM04) was processed with an algorithm programmed in python in order to obtain an estimation of the annual density of transport in the HSR, measured in terms of annual passengers over the complete infrastructure (total transport over the corridor divided by the length of the corridor). The algorithm and its results are gathered in SM05. The python script generates four “XX.csv” files (where XX is the name of the corridor), one for each corridor. Each .csv file has to be converted into .xlsx format, and the data inserted into the "XX_corridor_2016.xlsx" file, at cell "D6". The .xlsx file calculates minimum, maximum, mean and standard deviation values of possible passenger movements between each pair of stations. The .xlsx file also calculates a histogram for the density of transport. The parameters for the calculation of transport in each corridor are the boundary conditions provided by Galán et al. [3] and García [2] for the number of passengers entering or leaving each station (in thousands), and the data provided by Fundación de los Ferrocarriles Españoles [9] for the number of passengers in some specific connections (also in thousands). For each HSR corridor with n stations, characterised by its distance matrix ('distances', an *n* × *n* matrix with the distances between any pair of stations) and the annual travellers arriving or departing from each station (A, B, C, D…), the algorithm allows the random generation of an *n* × *n* matrix ('R') with the movements between any pair of stations, complying with the boundary conditions. For each calculation, a recursive algorithm is executed until total error (e) falls below a maximum value (2% for the Northern corridor, 4.1% for the Levante corridor, 1% for the Andalusia corridor and 5% for the Catalonia corridor). Multiple executions (10^5^) of the algorithm provides probabilities for passenger transport on the corridor under consideration to be statistically treated. Results are gathered in the SM06 file (“SM06.docx”).

The application of Eq. (1) to the primary and secondary data reported in Section 1 for each of the corridors is carried out, for each of the scenarios considered, in the spreadsheet tab (labelled with the name of the scenario: “BASELINE SCENARIO”, “S1”, “S2”, “S3”, “S4”, “S5”; the description of the scenarios is gathered in Table 1) from each of the Excel files included in SM01.

## CRediT Author Statement

**Andoni Kortazar:** Writing – original draft, Writing – review & editing, Investigation, Software; **Gorka Bueno:** Conceptualization, Writing – original draft, Methodology, Software; **David Hoyos:** Writing – review & editing, Supervision.

## Declaration of Competing Interest

The authors declare that they have no known competing financial interests or personal relationships that could have appeared to influence the work reported in this paper.
